# Structural Refinement of Proteins by Restrained Molecular Dynamics Simulations with Non-interacting Molecular Fragments

**DOI:** 10.1371/journal.pcbi.1004368

**Published:** 2015-10-27

**Authors:** Rong Shen, Wei Han, Giacomo Fiorin, Shahidul M. Islam, Klaus Schulten, Benoît Roux

**Affiliations:** 1 Department of Biochemistry and Molecular Biology, Gordon Center for Integrative Science, University of Chicago, Chicago, Illinois, United States of America; 2 Beckman Institute, University of Illinois at Urbana–Champaign, Urbana, Illinois, United States of America; 3 Institute for Computational Molecular Science, Temple University, Philadelphia, Pennsylvania, United States of America; George Mason University, UNITED STATES

## Abstract

The knowledge of multiple conformational states is a prerequisite to understand the function of membrane transport proteins. Unfortunately, the determination of detailed atomic structures for all these functionally important conformational states with conventional high-resolution approaches is often difficult and unsuccessful. In some cases, biophysical and biochemical approaches can provide important complementary structural information that can be exploited with the help of advanced computational methods to derive structural models of specific conformational states. In particular, functional and spectroscopic measurements in combination with site-directed mutations constitute one important source of information to obtain these mixed-resolution structural models. A very common problem with this strategy, however, is the difficulty to simultaneously integrate all the information from multiple independent experiments involving different mutations or chemical labels to derive a unique structural model consistent with the data. To resolve this issue, a novel restrained molecular dynamics structural refinement method is developed to simultaneously incorporate multiple experimentally determined constraints (e.g., engineered metal bridges or spin-labels), each treated as an individual molecular fragment with all atomic details. The internal structure of each of the molecular fragments is treated realistically, while there is no interaction between different molecular fragments to avoid unphysical steric clashes. The information from all the molecular fragments is exploited simultaneously to constrain the backbone to refine a three-dimensional model of the conformational state of the protein. The method is illustrated by refining the structure of the voltage-sensing domain (VSD) of the Kv1.2 potassium channel in the resting state and by exploring the distance histograms between spin-labels attached to T4 lysozyme. The resulting VSD structures are in good agreement with the consensus model of the resting state VSD and the spin-spin distance histograms from ESR/DEER experiments on T4 lysozyme are accurately reproduced.

## Author Summary

Knowledge of multiple conformational states of membrane transport proteins is a prerequisite to understand their function. However, the determination of atomic structures for all these states with conventional high-resolution approaches can be very challenging due to inherent difficulties in high yield purification of functional membrane transport proteins. Various complementary structural information of proteins in their native states can be obtained by a variety of biophysical and biochemical methods with site-directed mutations. Here, a novel restrained molecular dynamics structural refinement method is developed to help derive a structural model that is consistent with experimental data by incorporating all the experimental constraints simultaneously through the use of non-interacting all-atom molecular fragments. The method can be easily and effectively extended to incorporate many kinds of structural constraints from a variety of biophysical and biochemical experiments, and should be very useful in generating and refining models of proteins in specific functional states.

This is a *PLOS Computational Biology* Methods paper

## Introduction

Membrane proteins can access multiple conformational states that are of functional importance. Knowledge of the three-dimensional (3D) structure of all the relevant conformations in atomic detail is necessary to understand how these membrane proteins accomplish their function at the molecular level. Despite great progress, it is often a challenge to determine high-resolution protein structures under physiological conditions using conventional approaches, e.g., x-ray crystallography [1–4], cryo-electron microscopy [5, 6] and nuclear magnetic resonance (NMR) spectroscopy [7, 8] due to the inherent difficulties in protein expression, purification and stability. The task is even more difficult when the goal is to determine the structure of specific functional states because such determination is possible only if one is able to stabilize the respective states through the control of experimental conditions or by introducing ligands or mutations. Alternatively, a wealth of complementary structural information of proteins in their native states can be obtained by biophysical and biochemical approaches. For example, electron paramagnetic resonance (EPR) spectroscopy can estimate solvent accessibilities of spin-labeled sites on a protein [9–12], double electron-electron resonance (DEER) spectroscopy, a powerful pulsed electron spin resonance (ESR) technique, can determine distance histograms between pairs of spin-labels covalently bound to a protein [13, 14], and solid-state NMR spectroscopy can investigate the orientation of transmembrane (TM) helices of membrane proteins and peptides in lipid environments [15, 16]. When combined with computational modeling methods and molecular dynamics (MD) simulations, the structural information from such experiments can be used for the determination and refinement of atomic structures of proteins in specific conformational states [17–22]. However, translating experimental measurements into biologically meaningful and computationally efficient structural constraints is still quite challenging [20]. Residue-residue interactions derived from engineered salt or metal ion bridges are of particular interests as they can be translated directly into specific and simple spatial restraints, which can be incorporated into restrained MD simulations [23]. In particular, metal ion bridges (e.g., Cd^2+^, Zn^2+^ and Mg^2+^) can be easily formed between pairs of cysteines, histidines, as well as glutamates and aspartates when they are within atomic proximity [24, 25].

Studies of voltage-sensing domains (VSDs) in various ion channels provide a good illustration of the benefits from using this type of strategy [26–32], as salt or metal ion bridges can break and reform during the functional cycle of a working channel, avoiding trapping the protein in non-functional or distorted conformations due to their low strength [32]. The VSD structure is formed by a small bundle of four antiparallel TM helices, S1 to S4. A series of basic residues distributed along the helix S4 provide the “gating charge” that senses the changes in membrane potential. Upon depolarization, the VSDs rearrange from resting state into active state, triggering the opening of the central pore domain of the channel [33, 34]. The scenario that conveys most accurately the voltage-activated conformational change of the VSD is the “sliding helix” mechanism in which the S4 segment translates principally along its main axis while maintaining its helical conformations [35–37]. It is believed that during the voltage-activated transition, the basic residues along S4 form sequential salt bridge interactions with acidic residues located along S1–S3.

The x-ray crystal structures of the VSD in the active state have been determined for a few voltage-gated K^+^ [38, 39] and Na^+^ [40–42] channels. However, there is currently no atomic structure of the VSD of voltage-gated ion channels in the resting state. The only available crystal structure of the resting state VSD is that of the voltage-sensitive phosphatase from *C*. *intestinalis* (Ci-VSP), which is a distant homolog of the VSD of voltage-gated ion channels [4]. Using structural restraints derived from cysteine-cysteine Cd^2+^ bridges, Henrion et al. constructed one open and four different closed structures of the VSD of the *Shaker* potassium channel by Rosetta modeling and MD simulations [32]. Vargas et al. built and simulated several models of the VSD of the Kv1.2 channel in the resting state with experimentally determined salt and metal ion bridges, and the results of these restrained MD simulations were then used to generate a consensus model of the resting state conformation of the VSD, which is consistent with a wide range of available experimental data [23, 43] and displays a high degree of similarity to the atomic models of the resting state VSD constructed by different research groups with different methods [44–46]. In these two studies, the experimentally derived structural constraints, e.g., metal ion bridges, were imposed explicitly by performing proper site-directed mutations and adding specific bridging ions, instead of being represented as simplified interatomic distance restraints [34, 46]. However, each of these structural constraints was applied individually in independent models of the VSD. It was not possible to explicitly model all the structural constraints at once as one site of the protein might be involved in different engineered structural constraints and would cause unphysical steric clashes.

**Fig 1:**
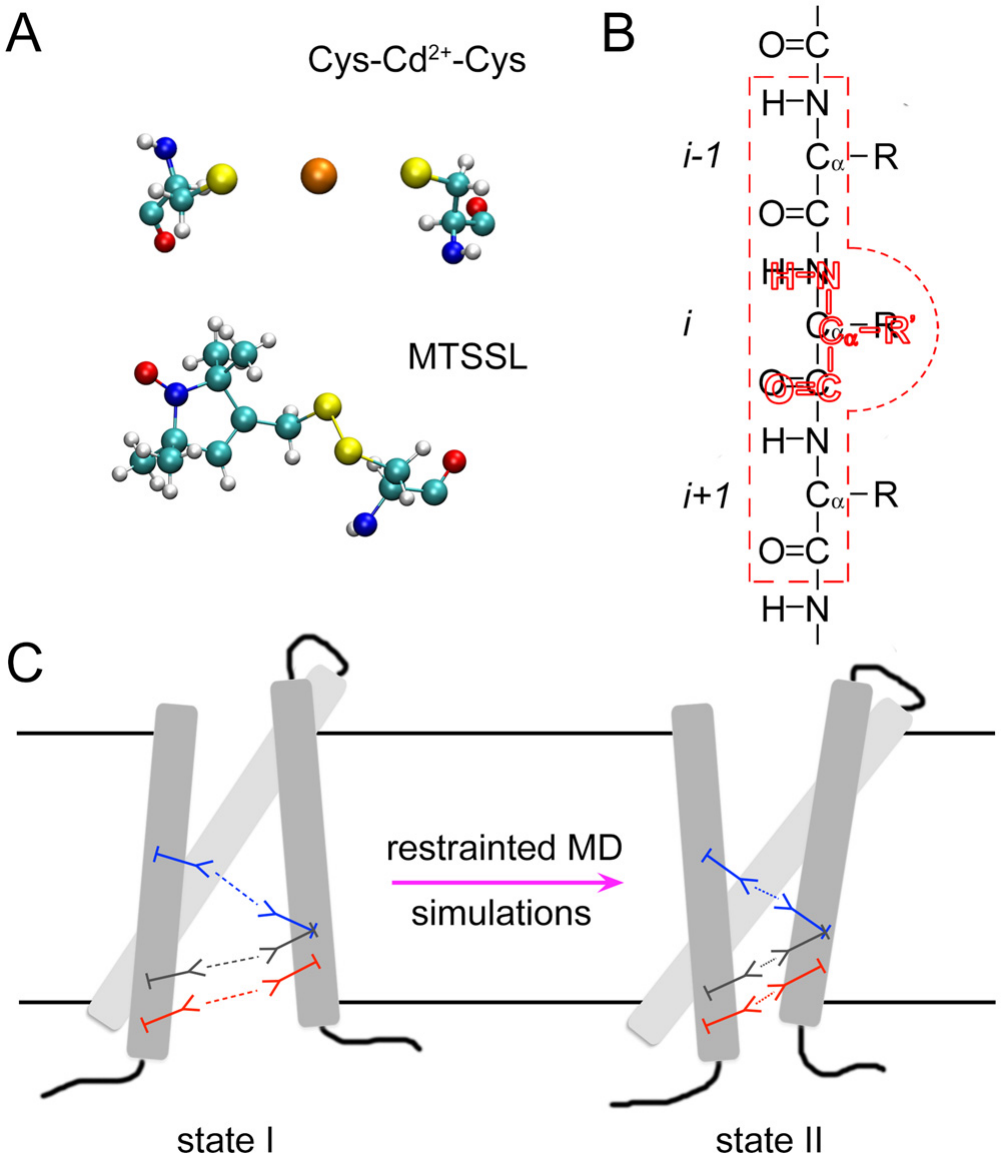
Schematic of the molecular fragments method. (A) Each structural constraint (e.g., a metal ion bridge or a spin-label) is present as a molecular fragment with all atomic details in the system. (B) The residue(s) in the molecular fragment (colored in red) is/are attached to the targeting residue(s) in the wild-type protein, via harmonic restraints, with their backbone atoms (N, C, O and C_*α*_) staying on top of each other, respectively. The residue in the molecular fragment does not have interactions with its targeting residue in the wild-type protein (residue *i*), and the interactions between the backbone atoms of the residue in the molecular fragment and the backbone atoms of the two nearby residues in the wild-type protein (residues *i*-1 and *i*+1; embraced by red dashed lines) are also turned off. (C) During the restrained MD simulations, the interactions within each molecular fragment are evaluated accurately triggering the conformational changes of the wild-type protein, while different molecular fragments do not have interactions with one another.

In the present study, we develop a novel structural refinement method by introducing the concept of non-interacting molecular fragments, which are used in the context of restrained MD simulations that can integrate the information from multiple experimental constraints simultaneously. The molecular fragments (e.g., metal ion bridges or spin-labels) are defined with all atomic details (Fig 1A). The fragments are anchored to the protein via their overlapping backbone atoms (N, C, O and C_α_) using harmonic restraints (Fig 1B). The interaction within each molecular fragment is treated realistically, while there is no interaction between different molecular fragments. To facilitate the refinement of atomic models of proteins, the molecular fragment method has been implemented in NAMD [47] that is efficient in MD simulations, and has been assisted further by a VMD [48] plugin designed to provide a user-friendly interface for constructing the models (S1 Fig). To test and illustrate the feasibility of the method, we refined the models of the resting state VSD of the Kv1.2 channel from Khalili-Araghi et al. [44] in an explicit lipid bilayer with multiple molecular fragments based on engineered salt and metal ion bridges between the four TM helices (S1–S4) of the VSD [28–32]. The VSD provides a good illustration for the present method because the 3D structure of the active state is known from crystallography, and experimental data must be used to determine the conformation of the resting state (Fig 1C). The results of the restrained MD simulations show that the structural restraints can be satisfied simultaneously without large conformational changes of the VSD from its starting configuration. The final structures are in good agreement with each other and with a previously established consensus model [23, 43], and are stable in subsequent simulations without the restraints. The non-interacting molecular fragment method has also been extended to incorporate the mean-field restrained-ensemble MD (reMD) simulation method with methanethiosulfonate spin-labels (MTSSL) as molecular fragments and the ESR/DEER distance histograms as structural constraints [49–51]. A total of 51 pairs of experimental distance histogram restraints were applied to 34 explicit spin-labels inserted at different positions in a single T4 lysozyme (T4L), and each spin-label was expanded into 25 copies. It is found that all the experimental distance histograms can be accurately reproduced simultaneously in the reMD simulations.

## Results and Discussion

**Fig 2:**
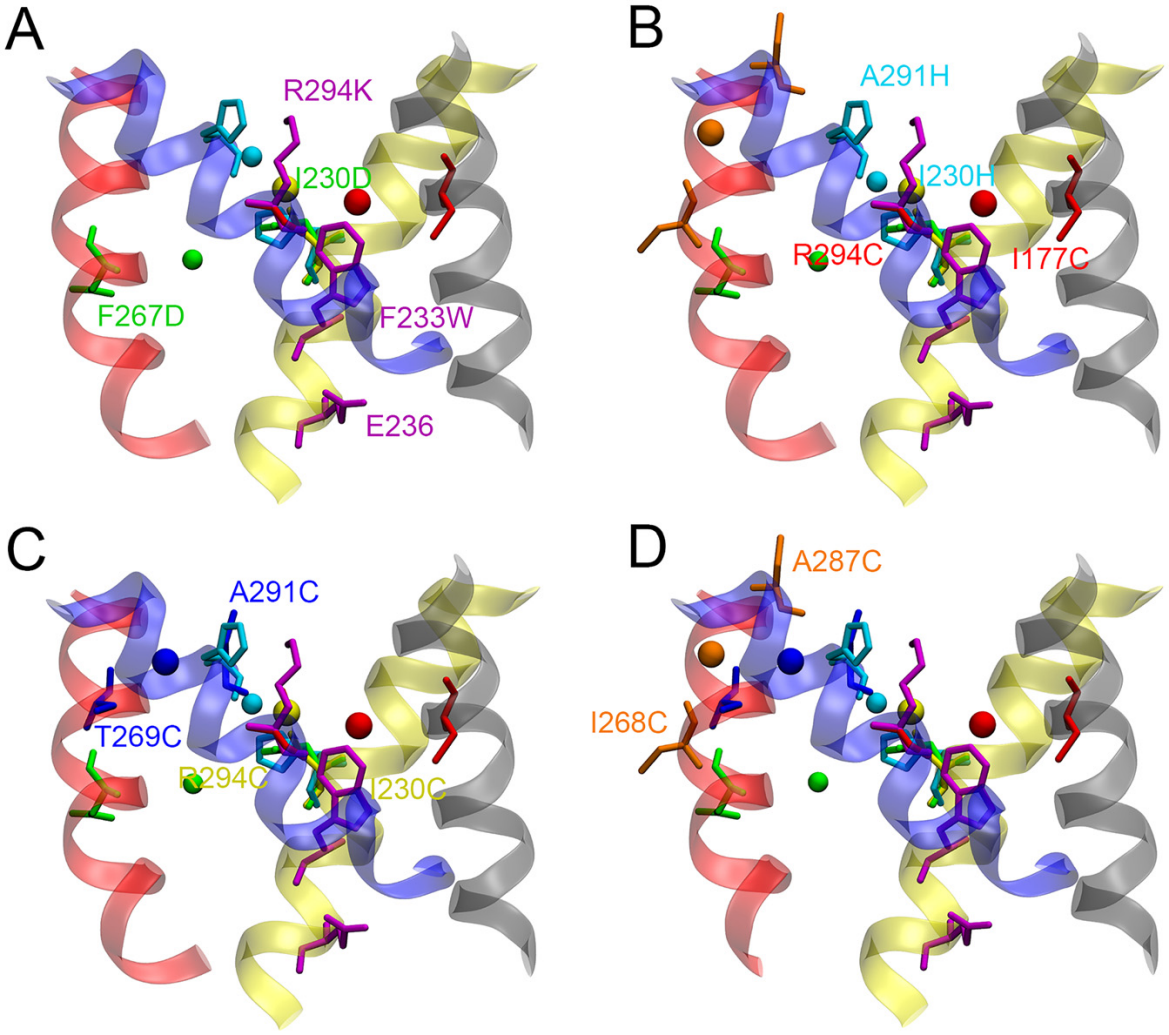
Starting configurations of the four models of the VSD with different molecular fragments. (A) model-1. (B) model-2. (C) model-3. (D) model-4. The four TM helices, S1 (gray), S2 (yellow), S3 (red) and S4 (blue), are represented in transparent ribbons. Molecular fragments, I177C–Cd^2+^–R294C, I230C–Cd^2+^–R294C, I230D–Mg^2+^–F267D, F233W/E236–R294K, I230H–Zn^2+^–A291H, I268C–Cd^2+^–A287C, and T269C–Cd^2+^–A291C, are colored in red, yellow, green, magenta, cyan, orange, and blue, respectively, with amino acids being represented in sticks and ions in spheres.

Four models of the resting state VSD of Kv1.2 with different structural constraints were generated (Table 1). In the first model (model-1), shown in Fig 2A, we simultaneously applied the first five structural constraints between S1 and S4, S2 and S4, and S2 and S3. Vargas et al. separately imposed these five structural constraints in their refinement of the resting state VSD of the Kv1.2 channel [23]. It was shown that each of the restraints could be accommodated by the VSD with little rearrangement of the backbone of S1–S4, and the requirement of the five interactions could all be satisfied in their final consensus structural model. As a result, we included the first five structural constraints in all of the four models. The last two structural constraints were reported recently [32], thus they were further incorporated separately or all at once in model-2 to model-4, in combination with the first five structural constraints, as a comparison.

**Table 1:** Details of the models of the VSD of the Kv1.2 channel.

Molecular fragment (MF)		Location	Model-1^a^	Model-2	Model-3	Model-4
1	I177C–Cd^2+^–R294C [28]	S1/S4	+	+	+	+
2	I230C–Cd^2+^–R294C [28]	S2/S4	+	+	+	+
3	I230D–Mg^2+^–F267D [29]	S2/S3	+	+	+	+
4	F233W/E236–R294K [30]	S2/S4	+	+	+	+
5	I230H–Zn^2+^–A291H [31]	S2/S4	+	+	+	+
6	I268C–Cd^2+^–A287C [32]	S3/S4		+		+
7	T269C–Cd^2+^–A291C [32]	S3/S4			+	+

^**a**^The “+” symbol means that the non-interacting molecular fragment was included in the modeling.

Practically, the heavy backbone atoms of the residue in the molecular fragment (colored in red in Fig 1B) are anchored to the respective heavy backbone atoms of the assigned residue in the VSD (residue *i* in Fig 1B). The interaction between the residue of the molecular fragment and its targeting residue (residue *i*) of the VSD, and the interaction between the backbone atoms of the molecular fragment residue and the backbone atoms of the two nearby residues of residue *i* (residue *i*-1 and residue *i*+1) are turned off. While the interactions within each molecular fragment are unaltered and treated realistically, the molecular fragments of the different constraints do not see one another (i.e., they are “non-interacting”) to avoid unphysical steric clashes.

In the initial structure of model-1, the distance between the C_*β*_ atoms of the two cysteine residues in the molecular fragment MF-1 (I177C–Cd^2+^–R294C) is 9.65 Å and the distances between the Cd^2+^ ion and the sulfur atoms of the cysteine residues are 5.10 Å. The corresponding C_*β*_-C_*β*_ and S_*γ*_-Cd^2+^ distances are 10.26 and 3.69 Å, respectively, in the molecular fragment MF-2 (I230C–Cd^2+^–R294C). The distances are too large for a Cys–Cd^2+^–Cys bridge, as the geometric restraint of a Cys–Cd^2+^–Cys bridge requires that the C_*β*_ atoms of the two cysteines be apart from each other by not more than 9 Å [32].

**Fig 3:**
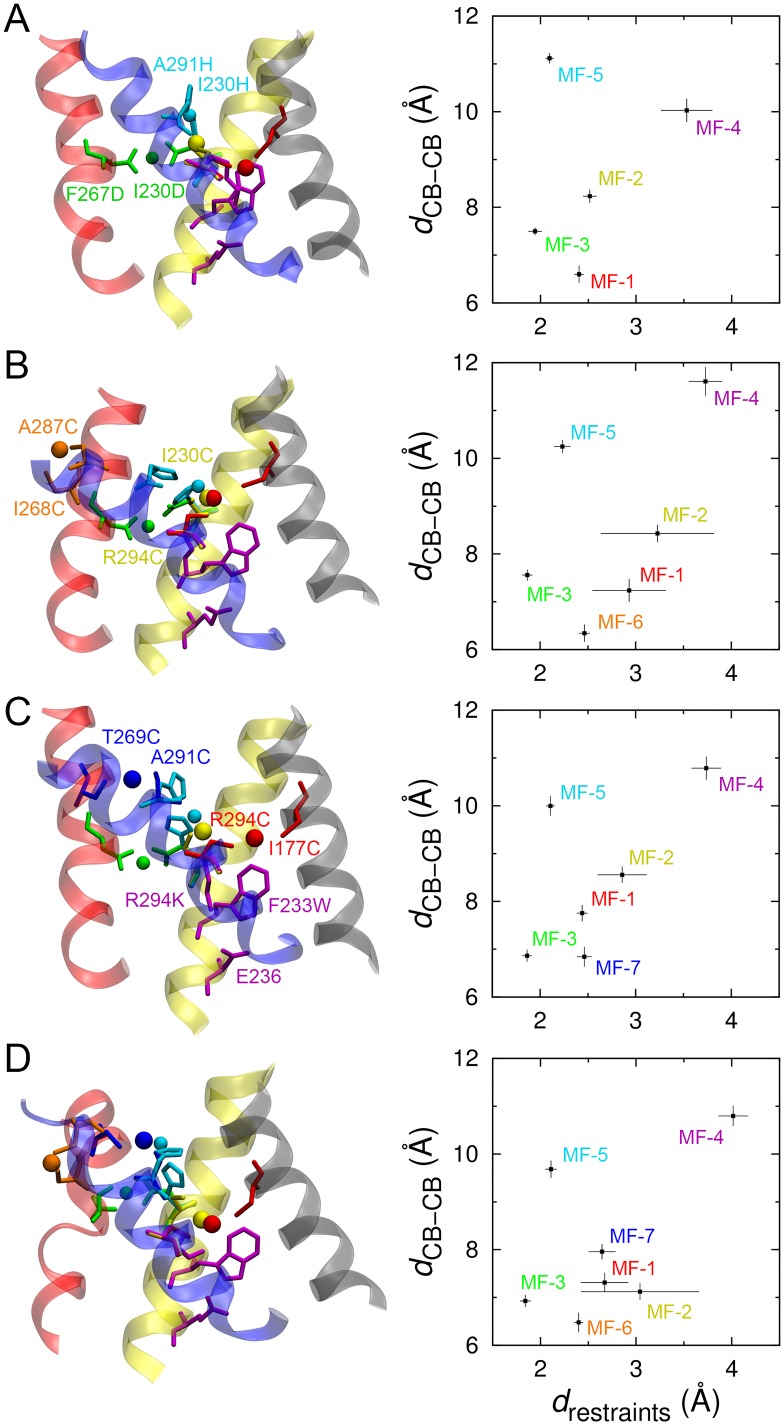
Final configurations of the four models of the VSD with different molecular fragments. The final configuration (left panel) and the correlation between the C_*β*_-C_*β*_ distance and the S_*γ*_-Cd^2+^, O_*δ2*_-Mg^2+^, N_*ε*_-Zn^2+^, or N_*ζ*_-O_*ε2*_ distance in each molecular fragment (right panel) of (A) model-1, (B) model-2, (C) model-3, and (D) model-4. The last 10 ns trajectories of the restrained MD simulations were used to calculate the average values and standard deviations of the distances.

**Fig 4:**
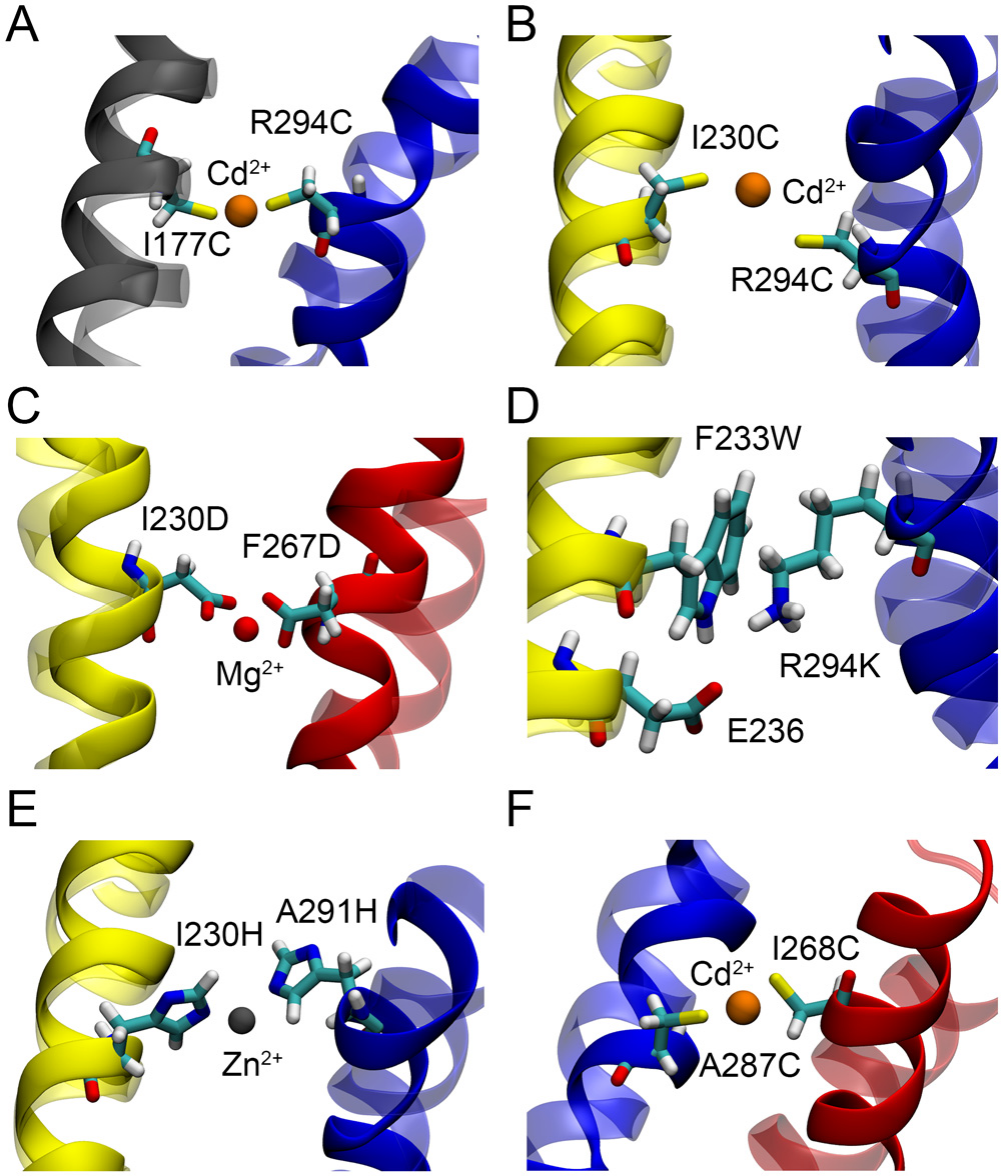
The final coordinates of the molecular fragments in model-3 after the restrained MD simulation. (A–F) The configurations of the four TM helices before and after the restrained MD simulation are shown in transparent and solid ribbons, respectively.

To improve the model, harmonic distance restraints between the Cd^2+^ ion and the S_*γ*_ atoms, centered at 2.6 Å [24], and harmonic angle restraints between S_*γ*_-Cd^2+^-S_*γ*_, centered at 180°, were applied in the restrained MD simulations containing cysteine-cysteine Cd^2+^ bridges (Table 2). To allow the VSD and the molecular fragments to adjust their conformations smoothly, the force constants for the harmonic restraints were gradually turned on during the simulations (S1 Table). The average distances between S_*γ*_ and Cd^2+^ and between C_*β*_ and C_*β*_ for MF-1 had been reduced to 2.40 ± 0.05 Å and 6.58 ± 0.19 Å, respectively, prior to the last 10 ns of the simulations. For MF-2, the average S_*γ*_-Cd^2+^ distance was 2.52 ± 0.07 Å, which is similar to that for MF-1, while the average C_*β*_-C_*β*_ distance was 8.24 ± 0.14 Å, 1.66 Å longer than that for MF-1. It is expected that the C_*β*_-C_*β*_ distance exhibits a wider spread than the ligand-metal (e.g., S_*γ*_-Cd^2+^, O_*δ*_-Mg^2+^ and N_*ε*_-Zn^2+^) and salt bridge (e.g., N_*ζ*_-O_*ε*_ and N_*ζ*_-O_*δ*_) distances due to the multiple rotameric states of the side chains of the interacting residues (Figs 3 and 4).

**Table 2:** Force field parameters for the metal ion bridges.

Bonds	*k*_b_ (kcal/mol/Å^2^)	*b*_0_ (Å)
S_*γ*_-Cd^2+^	10.0	2.6
O_*δ2*_-Mg^2+^	10.0	2.1
N_*ε*_-Zn^2+^	10.0	2.1
N-N, C_*α*_-C_*α*_,	50.0	0.0^a^
C-C, O-O		
Angles	*k*_θ_ (kcal/mol/rad^2^)	*θ*_0_ (degree)
S_*γ*_-Cd^2+^-S_*γ*_	10.0	180.0
O_*δ2*_-Mg^2+^-O_*δ2*_	10.0	180.0
C_*ε1*_-N_*ε*_-Zn^2+^	10.0	127.0
C_*δ2*_-N_*ε*_-Zn^2+^	10.0	125.5
Dihedrals	*k*_χ_ (kcal/mol/rad^2^)	*χ*_0_ (degree)
C_*γ*_-C_*δ2*_-N_*ε*_-Zn^2+^	10.0	180.0
Improper torsions	*k*_ϕ_ (kcal/mol/rad^2^)	*ϕ*_0_ (degree)
N_*ε*_-C_*δ2*_-C_*ε1*_-Zn^2+^	10.0	0.0
N_*ε*_-C_*ε1*_-C_*δ2*_-Zn^2+^	10.0	0.0

^a^The heavy backbone atoms (N, C, O and C_*α*_) of the patching residues in molecular fragments and the targeting residues in the protein overlap each other

In addition to the Cd^2+^ ion bridge, two other metal (Mg^2+^ and Zn^2+^) ion bridges were also considered in the models. In the molecular fragment MF-3 (I230D–Mg^2+^–F267D), the C_*β*_ atoms of the aspartate residues are initially 12.59 Å apart. The distances between the Mg^2+^ ion and the O_*δ2*_ atoms and the O_*δ2*_-Mg^2+^-O_*δ2*_ angle were harmonically restrained during the simulations, with an equilibrium bond distance of 2.1 Å [25] and an equilibrium angle of 180°, to form the aspartate-aspartate Mg^2+^ bridge (Table 2). The average C_*β*_-C_*β*_ and Mg^2+^-O_*δ2*_ distances as seen in the last 10 ns of the trajectory were 7.51 ± 0.08 Å and 1.94 ± 0.07 Å, respectively. It is also seen that the Mg^2+^-O_*δ2*_ distance is very stable in all of the four models (Fig 3), attributed to the strong bidentating interactions between Mg^2+^ and carboxylate groups of the aspartate residues [25]. For the histidine-histidine-Zn^2+^ bridge in the molecular fragment MF-5 (I230H–Zn^2+^–A291H), the imidazole nitrogen atom N_*ε*_ was assumed to coordinate the Zn^2+^ ion, as it appears to be the configuration with the highest propensity in the protein data base [24]. Furthermore, so-called improper torsion and dihedral restraints were applied to restrain the Zn^2+^ ion within the planes of the imidazole rings (Table 2). Harmonic restraints were applied to control the C_*ε1*_-N_*ε*_-Zn^2+^ and C_*δ2*_-N_*ε*_-Zn^2+^ angles, employing geometrical characteristics according to the crystal structures (e.g., PDB IDs: 1CBX, 1I4P, 1STE and 3CPA) containing the histidine-histidine-Zn^2+^ bridge [52–55]. Noting that the Zn^2+^ ion could also be bound by the N_*δ*_ atom, as observed in the crystal structures, we tried to restrain the Zn^2+^-N_*δ*_ bonds in our models and found that the Zn^2+^-to-N_*δ*_ distance could easily be satisfied, but that the orientation of the Zn^2+^-N_*δ*_ bonds with respect to the imidazole rings was hard to be maintained.

**Fig 5:**
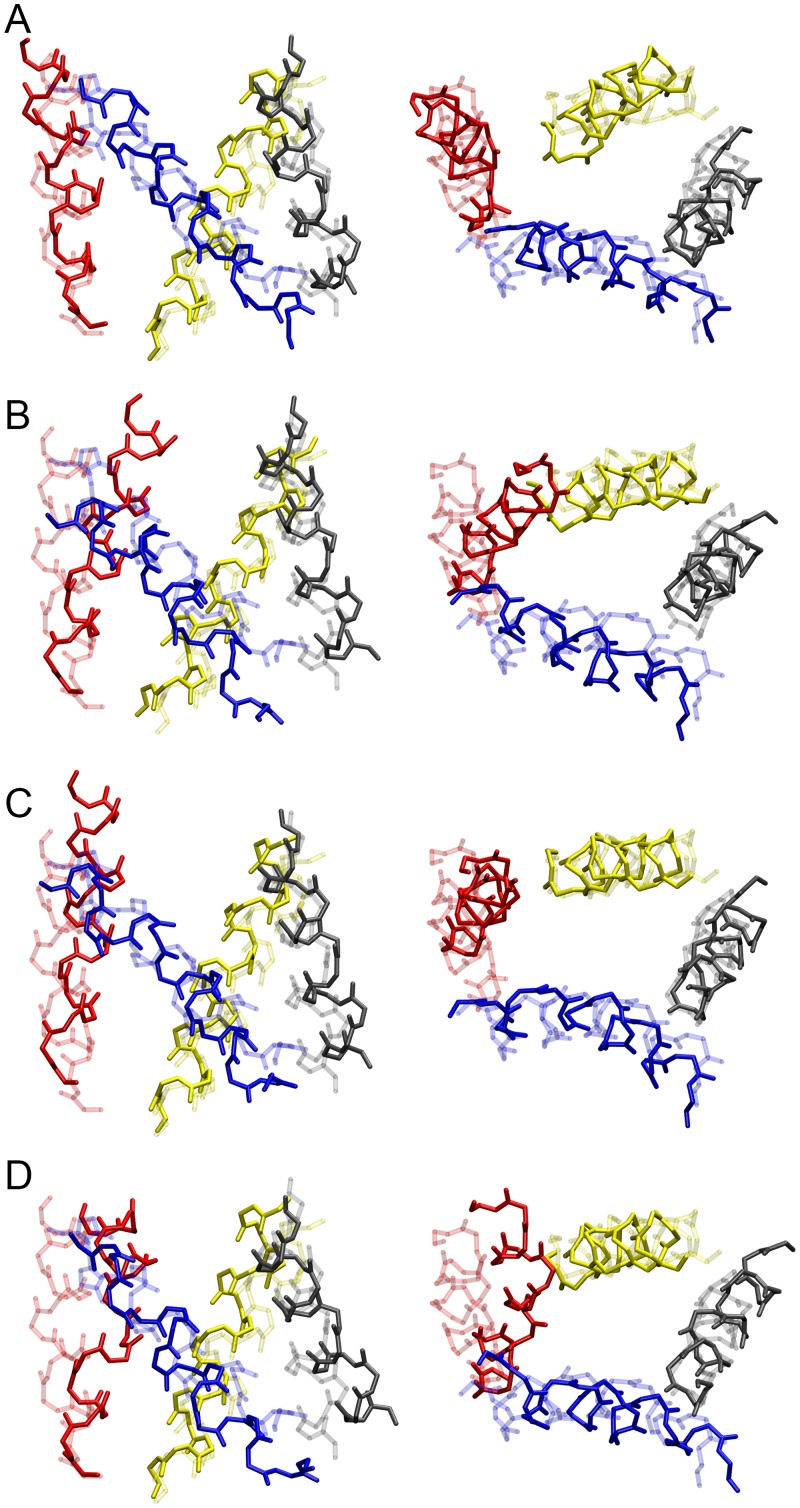
Superimposition of the initial and final configurations of the four models of the VSD. (A) model-1, (B) model-2, (C) model-3, and (D) model-4. The initial and final configurations of the backbone atoms are represented in transparent and solid sticks, respectively.

It has been shown that the double mutant F290W/R362K can further stabilize the resting state of the *Shaker* channel than the single mutant F290W [30]; apparently, the double mutant enables the formation of an electrostatic interaction between R362K and E293, which is one helix turn below F290W, in the resting state. To incorporate this lysine-glutamate salt bridge into the models of the VSD resting state of the Kv1.2 channel, we constructed a molecule fragment of F233W–R294K (MF-4). The amino group of the R294K side chain and the carboxylate group of the E236 side chain are initially far away from each other (further than 16 Å). To form the salt bridge, a harmonic restraint was applied between the N_*ζ*_ atom of R294K and the O_*ε2*_ atom of E236 with an equilibrium bond distance of 3.5 Å. The N_*ζ*_ atom moved towards the O_*ε2*_ atom quickly during the simulation and the average N_*ζ*_-O_*ε2*_ distance reduced to 3.72 ± 018 Å for the final 10 ns, with a decrease of the distance between the C_*β*_ atoms of E236 and R294K within roughly 5 Å. Superposition of the initial and final configurations of the VSD of model-1 using the STAMP structural alignment algorithm [56] reveals little rearrangement of the protein (Fig 5A). The backbone root-mean-square deviations (RMSDs) between the refined structure of model-1 with its initial structure and the consensus model, in the TM regions of the S1–S4 helices, are 2.2 Å and 3.1 Å (Table 3), respectively, indicating that the five residue-residue interactions applied by incorporating the proper molecular fragments into the model can be fulfilled simultaneously without large conformational changes of the protein backbone.

**Table 3:** The backbone RMSD values of the TM helices S1–S4 between different models.

	Model-1	Model-2	Model-3	Model-4	Khalili	Vargas	Jensen-A	Jensen-B	Jensen-C	Jensen-D
Model-1^a^	0.0	2.2	1.8	2.5	2.2	3.1	3.3	2.1	2.5	3.1
Model-2	2.2	0.0	1.7	1.7	2.7	3.0	3.2	3.1	2.6	3.8
Model-3	1.8	1.7	0.0	2.1	2.4	2.6	3.1	2.5	2.6	3.1
Model-4	2.5	1.7	2.1	0.0	2.9	2.8	3.3	3.2	2.8	3.6
Khalili	2.2	2.7	2.4	2.9	0.0	2.7	3.3	2.7	2.8	3.3
Vargas	3.1	3.0	2.6	2.8	2.7	0.0	2.7	2.7	2.6	2.8
Jensen-A	3.3	3.2	3.1	3.3	3.3	2.7	0.0	2.9	1.5	2.8
Jensen-B	2.1	3.1	2.5	3.2	2.7	2.7	2.9	0.0	2.3	3.1
Jensen-C	2.5	2.6	2.6	2.8	2.8	2.6	1.5	2.3	0.0	2.8
Jensen-D	3.1	3.8	3.1	3.6	3.3	2.8	2.8	3.1	2.8	0.0

^**a**^The RMSD values were calculated using the backbone atoms of the TM helices S1: residues 170–181, S2: residues 224–238, S3: residues 260–271, S4: residues 291–302 after performing a best-fit alignment between these models. The model of Jensen et al. [45] is a tetrameric channel having four subunits of the VSD (labeled A, B, C and D).

Two additional interactions between residues in the S3 and S4 helices, which have been recently found to arise in the resting state VSD of the *Shaker* channel [32], were further considered in our models. We incorporated another Cd^2+^ ion bridge molecular fragment MF-6 (I268C–Cd^2+^–A287C) and MF-7 (T269C–Cd^2+^–A291C) into model-2 and mode-3 (Fig 2), respectively, together with the initial five molecular fragments. As shown in Fig 3B, the average S_*γ*_-Cd^2+^ and C_*β*_-C_*β*_ distances of MF-6 had been reduced to 2.46 ± 0.06 Å and 6.34 ± 0.18 Å from 8.52 Å and 15.02 Å, respectively, for the final 10 ns. However, the average S_*γ*_-Cd^2+^ distances of MF-1 and MF-2 in model-2 are slightly larger (by ~ 0.5 Å) than the distance in model-1, with increased standard deviations, and the average C_*β*_-C_*β*_ distance of MF-4 is about 1.5 Å larger in model-2 than in model-1. The distance variations are consistent with the conformational rearrangement of the model. Comparison of the starting and final conformation of model-2 reveals an inward movement of S4, and an outward translation and a clockwise rotation (top view) of S3, while the S1 and S2 helices mainly retain their conformation (Fig 5B). The inward movement of S4, which resulted from the newly applied structural restraint MF-6, changed the configuration of the molecular fragments MF-1, MF-2 and MF-4, restraining the relative positions between S1 and S4, and S2 and S4. Similar conformational changes were observed for model-3 (Fig 5C), using the molecular fragment MF-7 to regulate the interaction between residues in S3 and S4. However, the S3 helix moved further outward and the S4 helix moved less inward in model-3 than in model-2, and the residue-residue interactions in the molecular fragments were well fulfilled (Figs 3C and 4). The two structural restraints (MF-6 and MF-7) were both applied in our last model, model-4, (Fig 2D). It was found that the S3 helix kinked in the center at the end of the simulation, although the structural restraints of MF-6 and MF-7 were satisfied (Figs 3D and 5D). We propose that the resides involved in the last two molecular fragments connecting the S3 and S4 helices are too close to each other, and the S3 helix is not able to adjust its conformation to satisfy the two structural constraints at the same time while maintaining an alpha-helical structure. However, they can be satisfied separately with slight conformational changes of the VSD as shown in model-2 and model-3. The RMSDs of the backbone atoms in the TM region of the S1–S4 helices between the four refined models and the models of Khalili-Araghi et al. [44], Vargas et al. [23] and Jensen et al. [45] lie within 1.7–3.8 Å of one another (Table 3), revealing a high degree of similarity between these structures at the atomic level [43]. In addition, the conformation of the four refined models remain stable during an unbiased MD simulation of 100 ns without the structural restraints (see S2 Fig), implying that the structural constraints will not displace the conformation of the VSD away from its “native” state(s). The Protein Data Bank (PDB) coordinates of the TM region of the refined model-3 are provided in Supplementary Material.

The present methodology with multiple non-interacting molecular fragments can also be used in the treatment of DEER data. DEER spectroscopy is a powerful electron spin resonance technique allowing the determination of distance histograms between pairs of spin-labels attached to a protein in their native environment. Recently, a mean-field restrained-ensemble molecular dynamics (reMD) simulation method was introduced to exploit ESR/DEER distance histograms in protein structural refinement [49–51]. The method was built on the framework of the multiple-copy locally enhanced sampling method, in which each spin-label side chain was expanded into *N* copies, yielding a total of *N* ^2^ spin-spin distances for each pair of spin-labels. Energy restraints were then implemented to couple the ensemble of copies of the spin-labels and enforce the experimental ESR/DEER distance histograms. In the reMD simulations, the different copies of a given spin-label do not have interactions with one another while the copies from different spin-labels can interact with each other. It requires that the spin-labels should be kept apart with certain distances from one another, downgrading the efficiency of the reMD simulation method. For example, up to 51 pairs of spin-spin distance histograms have been measured for a small benchmark protein of 165 residues, T4L, involving a total of 34 different site-directed spin-labeling sites. To apply all of the 51 experimental restraints simultaneously, four separate copies of the protein T4L were included in the simulation system to prevent steric clashes between the spin-labels [50].

**Fig 6:**
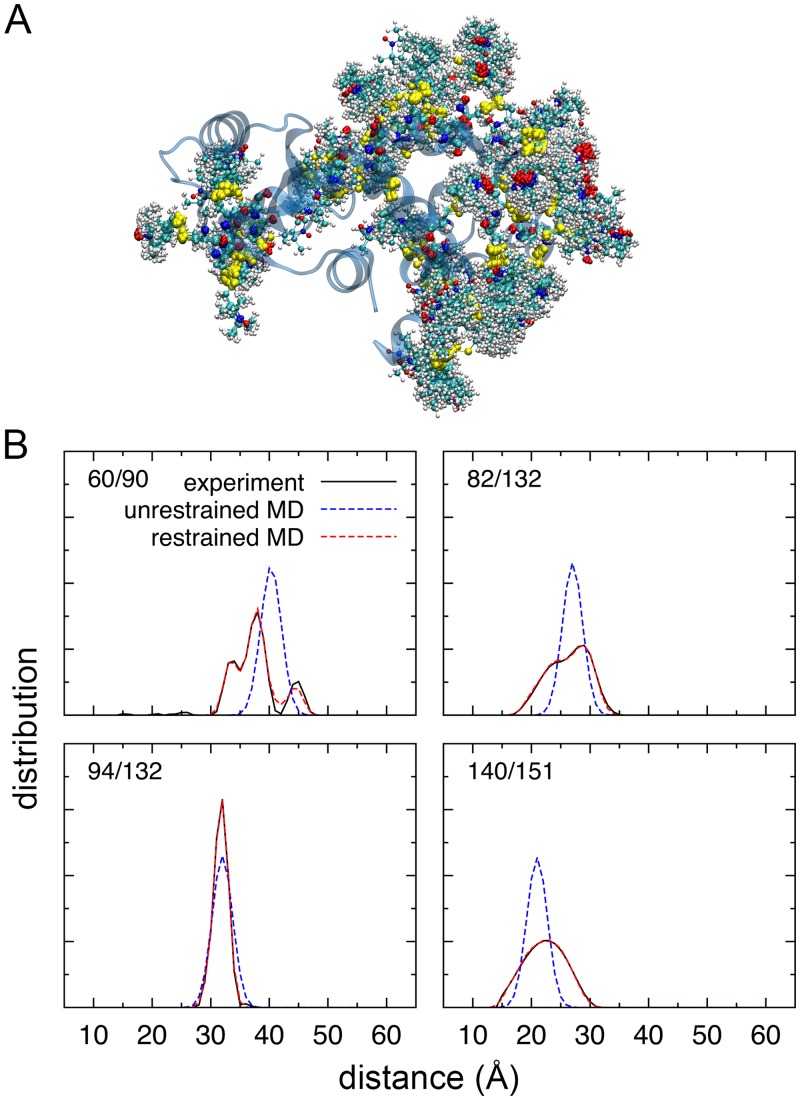
Configurations of the spin-labels attached to T4 lysozyme. (A) A snapshot of the T4 lysozyme system with 34 MTSSL spin-labels. The spin-label at each site has 25 copies and highlighted in the ball-and-stick representation. (B) Distance histograms of four pairs of spin-labels from ESR/DEER experiments (black solid line), and the molecular dynamics simulations without (blue dashed line) and with (red dashed line) an energy restraint. Please see the Supplementary Material, S3 and S4 Figs for the other 47 distance distributions.

The non-interacting molecular fragment method makes it possible to include all of the spin-labels together in a single copy of the protein in reMD simulations. As shown in Fig 6A, spin-labels were introduced at 34 positions in T4L, and the spin-label at each site has 25 copies. The 51 distance distribution data from DEER were then simultaneously used in the reMD simulation [49], which enforce an energy restraint to match the calculated distance distribution to agree with those from DEER (Fig 6B). All the 51 distance distributions data are shown in the Supplementary Material, S3 and S4 Figs. It should be noted that the unbiased reMD simulation without any restraint was not able to converge accurately toward the experimental distance histograms, especially for those histograms having complex structures (S3 and S4 Figs), as discussed in the previous studies [49, 50]. It is worth mentioning that the present molecular fragment method can also be used in a simpler reMD treatment of DEER data in which the spin-labels are represented by dummy spin-labels [49–51]. The reMD simulation of the dummy spin-label attached to T4L was also found to rapidly match the distance distribution with those obtained from EPR/DEER within a few ns (data not shown).

In summary, the atomic resolution structures of membrane transport proteins in different states are critical for developing an understanding of the proteins’ biological function at the molecular level. However, the determination of the high-resolution structures using conventional structure determination methods encounters different types of challenges and is often not feasible, especially for membrane proteins. We have developed a novel method based on non-interacting molecular fragments with restrained molecular dynamics to refine protein structures using information from residue-residue interactions derived from a wide range of biophysical and biochemical experiments. The multiple independent structural constraints are incorporated simultaneously on the basis of individual non-interacting molecular fragments represented in atomic detail. Using the new method, we have refined the atomic resolution structure of the VSD of the Kv1.2 channel in the resting state based on different sets of experimental restraints. A number of models were generated using different subset of experimental constraints. The method could efficiently generate a 3D structural model of the VSD that satisfies all the imposed experimental constraints simultaneously. The final refined models are quite similar to the Vargas et al. consensus model [23, 43] with the backbone RMSD between the final models and the consensus model, in the TM region of the S1–S4 helices, seen to lie between 2.6 and 3.1 Å. Extensions and applications of the molecular fragment method to incorporate other advanced simulation methods and structural constraints can also be achieved easily and conveniently. An illustrative test of the spin-spin distance distribution of T4L shows that the molecular fragment method is compatible with the mean-field reMD simulation method by treating each copy of the spin-labels as an individual molecular fragment. The resulting distance histograms of 51 pairs of spin-labels from a single copy of the protein accurately matched the experimental data simultaneously by applying a novel energy restraint to the ensemble of spin-labels.

## Materials and Methods

### Molecular fragment method

The molecular fragment method refines the VSD structural models by applying in the same simulations different structural constraints derived from individual experiments [28–32]. Each structural constraint corresponds to a molecular fragment containing a salt bridge or a metal ion bridge that connects two remote residues of the VSD. These molecular fragments were represented with all atomic details (Fig 1) and attached to assigned residues of the VSD by keeping the backbone atoms (N, C, O and C_α_) of the residues in the molecular fragments and their targeting residues in the VSD staying on top of each other through harmonic restraints centered at 0 Å. The bridging metal ions were placed at the centers of the fragments, coordinating to the S_*γ*_ atom of cysteine, the N_*ε*_ atom of histidine, the O_*δ2*_ atom of aspartate or the O_*ε2*_ atom of glutamate depending on types of fragments (S1 Movie).

For the sake of simplicity, the cysteine, histidine, aspartate and glutamate residues involved metal ion bridges were assigned in the deprotonated state carrying a negative charge [23], such that each molecular fragment is electrically neutral. We have built a VMD plugin to introduce the molecular fragments to the model and generate input files for the subsequent MD simulations (S1 Fig). This plugin is available in the Supplementary Material and at (www.ks.uiuc.edu/Research/MolFragReMD/).

All simulations containing molecular fragments were performed with NAMD [47]. To avoid steric clashes when simulating multiple molecular fragments, NAMD was modified such that any interaction between the fragments themselves was removed (“switched off”) during simulations. As heavy backbone atoms of the fragments were forced to stay on the top of the respective atoms of the targeting residues of the protein, the interactions between the overlapping parts of the fragments and the protein atoms were also removed (Fig 1). To further avoid any potential bad contact, an effective distance, $r_{ij}^{'}\;=\;\sqrt{r_{ij}^{2}+\Delta }$, was introduced to NAMD to calculate the interaction of pairs of atoms between the molecular fragments and the protein, where Δ is an input parameter chosen by the user. With this effective distance, unphysical small distances between pairs of atoms are allowed to occur without introducing abrupt fluctuations in the atomic forces that destabilize the simulations.

A technical issue associated with molecular fragment simulations is how to eliminate electrostatic interactions between different fragments. When using the particle mesh Ewald (PME) method [57], long-range electrostatic forces are calculated in reciprocal space. If the partial charges of all the fragments were all accumulated on the PME grid, the long-range electrostatic interactions between two fragments would be difficult to subtract in a pairwise manner. Because the fragments are restrained around the target residues of the protein, the latter are instead a good approximation for calculating long-range interactions. In this approach, only the protein’s charges are accumulated on the PME grid when calculating long-range electrostatic forces, while the fragments interact with their surroundings with truncated Coulomb and van der Waals potentials.

### Construction of the starting models

The structure of a single VSD (residues: 158–308) of the Kv1.2 channel in the resting state proposed by Khalili-Araghi et al. [44], subsequently assembled from the atomic model of Pathak et al. [22], was used to generate our starting models with VMD [48]. Here, seven non-interacting molecular fragments (labeled MF-1 to MF-7) were considered to model the resting state of the VSD: MF-1 is a cysteine-cysteine Cd^2+^ bridge involving residues I241C (S1) and R362C (S4) in *Shaker* [28]; corresponding residues in the Kv1.2 channel are I177C (S1) and R294C (S4). MF-2 is a cysteine-cysteine Cd^2+^ bridge involving residues I287C (S2) and R362C (S4) in *Shaker* [28]; corresponding residue in Kv1.2 are I230C (S2) and R294C (S4). MF-3 is an aspartate-aspartate Mg^2+^ bridge involving residues I287D (S2) and F324D (S3) [29], equivalent to I230D (S2) and F267D (S3) in the Kv1.2 channel. MF-4 is a lysine-glutamate salt bridge involving F290W (S2), R362K (S4) and E293 (S2) in *Shaker* [30]; corresponding residue in Kv1.2 are F233W (S2), R294K (S4) and E236 (S2). MF-5 is a histidine-histidine Zn^2+^ bridge involving residues I287H (S2) and A359H (S4) in *Shaker* [31]; corresponding residues in Kv1.2 are I230H (S2) and A291H (S4). MF-6 is a cysteine-cysteine Cd^2+^ bridge involving residues I325C (S3) and A355C (S4) in *Shaker* [32]; corresponding residue in Kv1.2 are I268C (S3) and A287C (S4) in the Kv1.2 channel. MF-7 is a cysteine-cysteine Cd^2+^ bridge involving residues T326C (S3) and A359C (S4) in *Shaker* [32]; corresponding residue in Kv1.2 are T269C (S3) and A291C (S4). In the case of MF-4, the double mutant F290W/R362K supposedly enables an electrostatic interaction between R362K and E293 in the *Shaker* channel. Four models of the VSD in the resting state were generated by considering various combinations of these interactions (Table 1). The histidine residues were modeled in a protonation state with a positive charge, and all other ionizable residues were assigned in their default ionization state, i.e., Glu and Asp carrying a negative charge, Lys and Arg carrying a positive charge, as in the atomic models of Khalili-Araghi et al [44]. The VSD was then embedded into a pre-hydrated 1-palmitoyl-2-oleoyl-sn-glycero- 3-phosphocholine (POPC) lipid bilayer. The membrane normal axis was aligned along the *z*-axis with the center of the membrane at *z* = 0. An additional sodium ion was inserted into the bulk water to make the system in an electrically neutral state.

To simulate DEER data, the x-ray crystal structure of T4L (PDB ID: 2LZM) was used to generate the starting T4L system with VMD [48]. The protein was solvated into a cubic water box with 8 chloride ions. The ionizable residues of T4L were assigned in their default ionization state and the final system is electrically neutral.

### Restrained molecular dynamics simulations

The protein, phospholipids and ions were described with the CHARMM36 force field [58–60], and water molecules with the TIP3P model [61]. Harmonic restraints with soft force constants were applied to maintain the conformation of the metal bridges [24, 25]. Harmonic restraints were also applied to the heavy backbone atoms (N, C_*α*_, C and O) of the molecular fragments and the corresponding atoms of their targeting residues (Table 2).

Following 5000 steps of energy minimization, each system was initially equilibrated for 1 ns with the protein and the molecular fragments being restrained to fill the gap between the protein and the lipids. The systems were then simulated for 20 ns with the harmonic restraints applied to the metal bridges being gradually increased. Secondary structure restraints were also applied to the four TM helices of the VSD to avoid large distortions of the protein at the initial stages of the refinement, and the force constant was gradually decreased. Another 80 ns simulation was carried out for each system with constant restraints to test the stability of the refined VSD and the molecular fragments (S1 Table). The refined systems were further simulated for 100 ns without the structural constraints, and the backbone atoms of the protein were restrained during the first 10 ns using a force constant of 1 kcal/mol/Å^2^.

A time step of 2 fs was used with the bonds involving hydrogen atoms being constrained using the SHAKE algorithm [62]. Electrostatic interactions were calculated using the PME method [57] with a grid density of at least 1 bin per Å^3^, and the van der Waals interactions were smoothly switched off after 10 Å and truncated beyond 12 Å. Periodic boundary conditions were imposed in all directions. The temperature of the systems was controlled at 300 K using the Langevin dynamics and the pressure was kept at 1 atm using the Nose-Hoover Langevin piston method [63, 64].

### Restrained-ensemble molecular dynamics (reMD) simulations

Spin-labels, consisting of disulfide bond linked MTSSL (1-oxyl-2,2,5,5-tetramethylpyrroline-3-methyl methanethiosulfonate) and cysteine, were attached at 34 sites of T4L using the VMD plugin we built. The spin-label at each site has 25 copies, and each of the copies was assigned as a separate molecule fragment. The heavy backbone atoms (N, C, O and C_*α*_) of the spin-labels and those of the targeting residues of T4L were forced to stay on top of each other, respectively, through harmonic restraints with a force constant of 10 kcal/mol/Å^2^ and an equilibrium distance of 0 Å. The interactions between the spin-labels and the rest of the system were scaled by 1/25. Because of the coding design of NAMD, one can only define at most 255 non-interacting molecular fragments, while there are a total of 850 copies (34×25) of spin-labels in the T4L system. To circumvent this constraint, several spin-labels that were separated by a distance larger than the non-bonded cutoff were grouped in the same molecular fragment. For example, the *s*-th copy of spin-label at site 59 and the *s’*-th copy of spin-label at site 128 could be classified into the same molecular fragment, while the *s*-th and *s’*-th copies of spin-label at site 59 or 128 were classified into two different molecular fragments, thus each copy of the spin-labels has no interactions with one another.

The distance histogram for each pair of spin-labels was restrained toward the experimental data from ESR/DEER using an ensemble energy restraint introduced by Roux and coworkers [49, 50]

$$U_{\text{RE}}=\frac{1}{2}K\sum_{\left\{\text{pair }ij\right\}}\sum_{\left\{bin\;n\right\}}{\left({\bar{h}}^{ij}\left(n\right)-H^{ij}\left(n\right)\right)}^{2}$$

where ${\overset{-}{h}}^{ij}(n)$ and *H* ^*ij*^(*n*) are the *n*-th bin of the histogram for the *ij* pair of spin-labels from the reMD simulations and experiments, respectively. A smooth differentiable Gaussian of width *σ* was used to construct the histograms

$${\bar{h}}^{ij}\left(n\right)=\frac{1}{N^{2}}\sum_{s=1}^{N}\sum_{S^{\prime }=1}^{N}\frac{1}{\sqrt{2\pi \sigma^{2}}}e^{-{\left(n\Delta r-\left|r_{i}^{s}-r_{j}^{s^{\prime }}\right|\right)}^{2}/2\sigma^{2}}$$

where *N* is the number of copies of a given spin-label, Δ*r* is the bin width, $\left|r_{i}^{s}-r_{j}^{s'}\right|$ is the distance between the oxygen atoms of the *s*-th copy of spin-lable *i* and the *s’*-th copy of spin-lable *j*. The histograms from the simulations and experiments were properly normalized, namely,

$$\sum_{\left\{\text{bin }n\right\}}{\bar{h}}^{ij}\left(n\right)\Delta r=\sum_{\left\{\text{bin }n\right\}}H^{ij}\left(n\right)\Delta r=1$$

for all the *ij* pair of spin-labels. The energy restraint has been introduced into NAMD under the collective variables (colvars) module. The force constant *K* was set to 500 kcal/mol/Å^2^, and the natural spread *σ* of the Gaussian was set to 1.1 Å. The bin width Δ*r* was set to 1 Å and *n* = 1, …, 60. The backbone of the T4L was harmonically restrained with a force constant of 100 kcal/mol/Å^2^ to prevent any large displacement and distortion.

The system was energy minimized and equilibrated for 2 ns without the energy restraint, followed by a 1 ns production run with the energy restraint. The simulations were performed in the NPT ensemble with a temperature of 300 K and a pressure of 1 atm using the Nose-Hoover Langevin piston method [63, 64]. The time step of the simulations was set to 1 fs and the bonds involving hydrogen atoms were constrained with the SHAKE algorithm [62]. The PME method was used to calculate the electrostatic interactions [57], and the van der Waals interactions were smoothly switched off from 10 to 12 Å. For the sake of completeness, the reMD simulation was also carried out using dummy spin-labels according to the previously established protocol [49–51].

All the features stated above, including the molecular fragment method and the distance histogram restraints, have been implemented in a modified version of NAMD. The source code and a brief tutorial of the molecular fragment method are available at (www.ks.uiuc.edu/Research/MolFragReMD/).

## Supporting Information

S1 Fig The interface of the VMD plugin used for constructing models with multiple molecular fragments. (TIF)

S2 Fig The trajectories of the backbone RMSDs of the TM region of the four VSD models as a function of simulation time and the superimposition of the starting and final configurations of the models in the extended MD simulations without the structural constraints. (TIF)

S3 Fig The distance histograms (h1–h28) for the spin-labels attached to T4 lysozyme from the ESR/DEER experiments and the mean-field restrained-ensemble simulations. (TIF)

S4 Fig The distance histograms (h29–h51) for the spin-labels attached to T4 lysozyme from the ESR/DEER experiments and the mean-field restrained-ensemble simulations. (TIF)

S1 Table Restraints applied to the metal bridges and the four transmembrane helices. (PDF)

S1 Movie Animation showing the process of constructing models with molecular fragments. (MPG)

S1 PDB Backbone of the S1–S4 helices of the refined model-3 for the voltage-sensing domain of Kv1.2. (PDB)

S1 VMD-Plugin Scripts and usage of the VMD plugin used for constructing models with multiple molecular fragments. (ZIP)
