# Supplementary figures and images for: Structural Refinement of Proteins by Restrained Molecular Dynamics Simulations with Non-interacting Molecular Fragments
Source: PLoS Comput Biol. 2015 Oct 27;11(10):e1004368. doi: 10.1371/journal.pcbi.1004368 (PMC4624691; doi:10.1371/journal.pcbi.1004368)

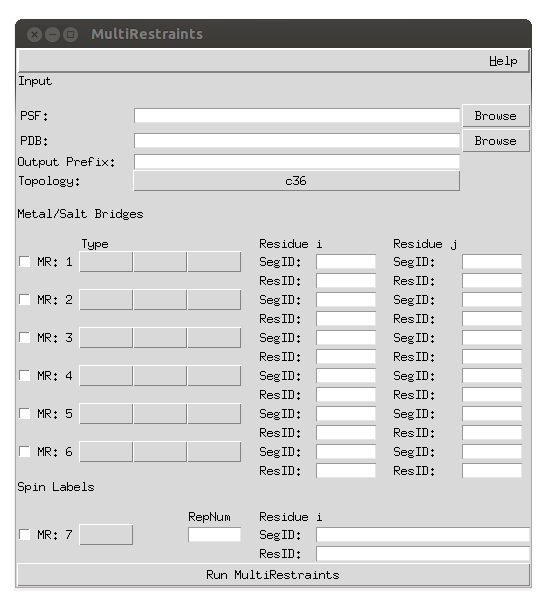

Supplement: S1 Fig — (TIF) [file pcbi.1004368.s001.tif]

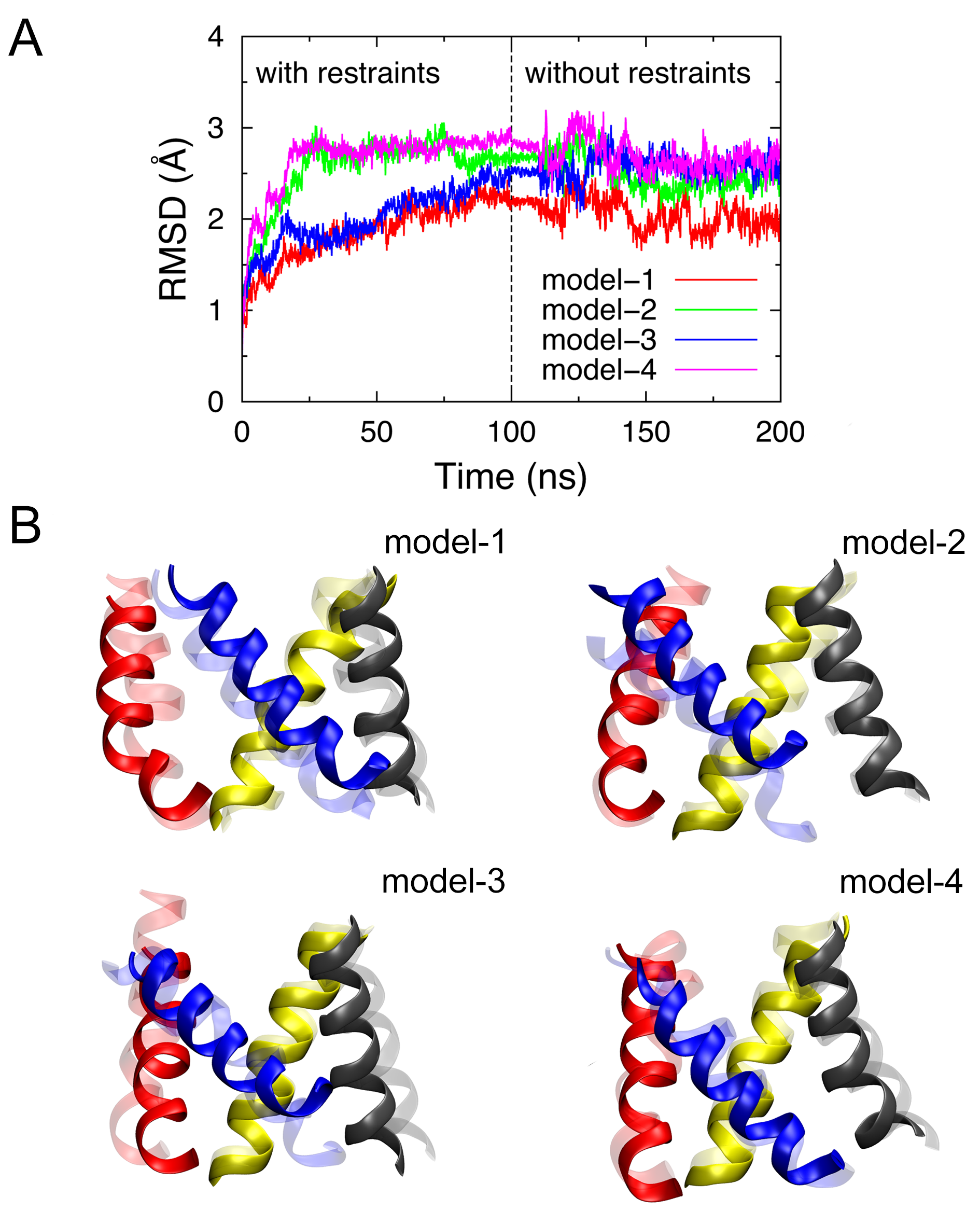

Supplement: S2 Fig — (TIF) [file pcbi.1004368.s002.tif]

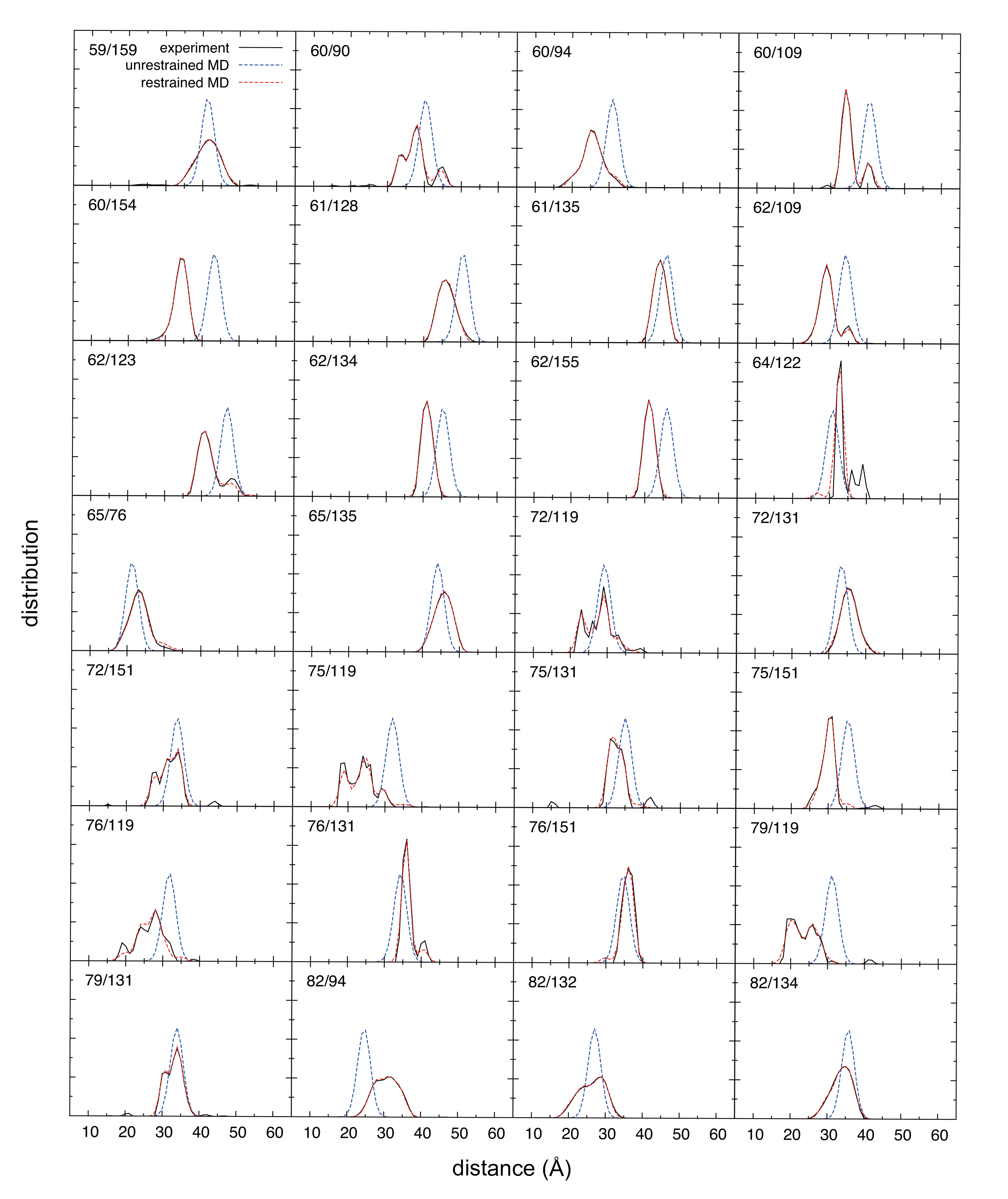

Supplement: S3 Fig — (TIF) [file pcbi.1004368.s003.tif]

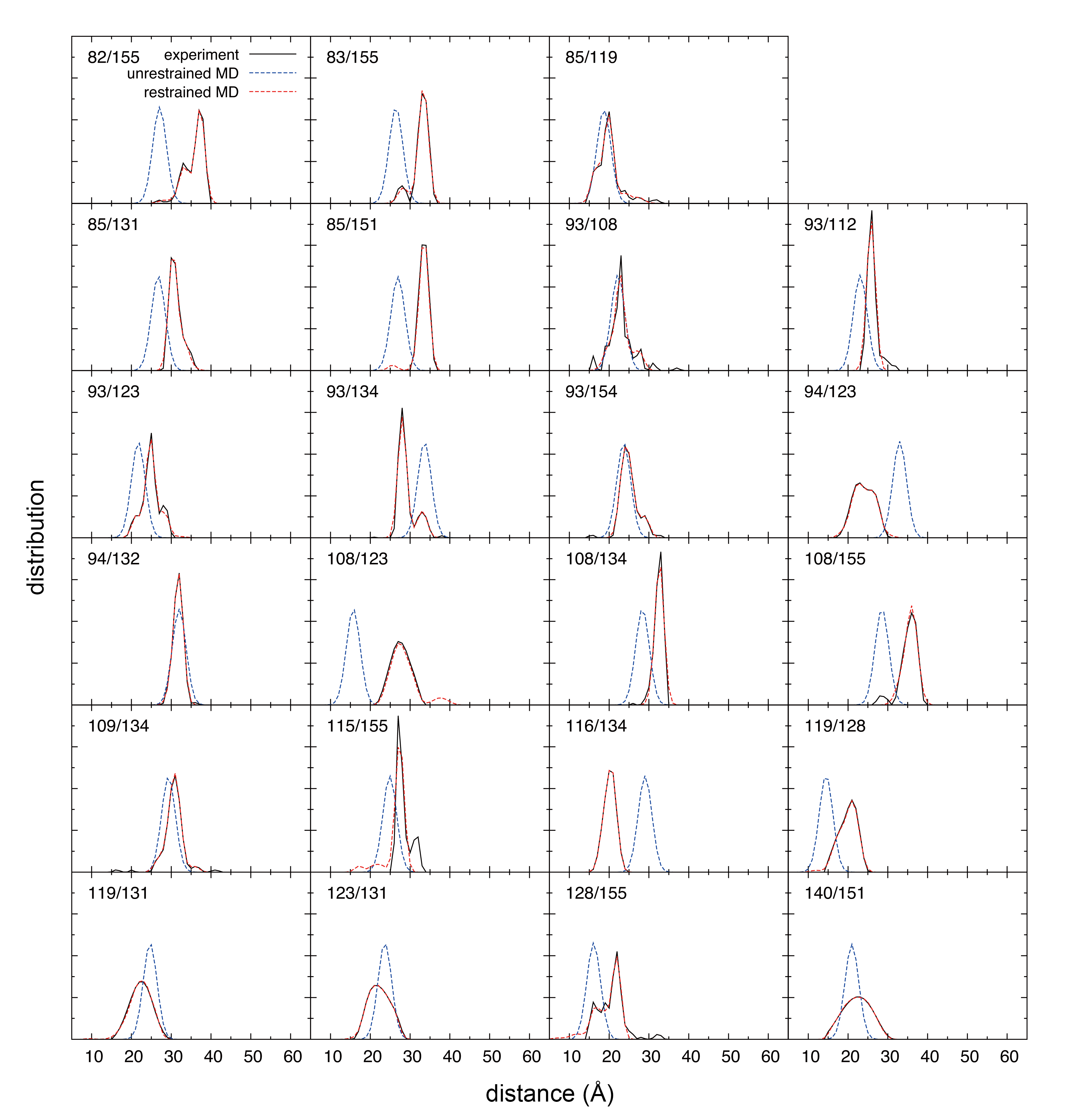

Supplement: S4 Fig — (TIF) [file pcbi.1004368.s004.tif]
