# Supplementary material for: Structural Refinement of Proteins by Restrained Molecular Dynamics Simulations with Non-interacting Molecular Fragments
Source: PLoS Comput Biol. 2015 Oct 27;11(10):e1004368. doi: 10.1371/journal.pcbi.1004368 (PMC4624691; doi:10.1371/journal.pcbi.1004368)
Supplement: S1 Table — (PDF) [file pcbi.1004368.s005.pdf]

Table S1. Restraints applied to the metal bridges and the four transmembrane helices (secondary structure; S1: residues 170–184, S2: residues 224–238, S3: residues 260–275, S4: residues 284–305) were gradually changed.

	Metal-ligand bonds (kcal/mol/Å^2)	Backbone atoms bonds (kcal/mol/Å^2)	Angles (kcal/mol/rad^2)	Dihedrals (kcal/mol/rad^2)	Improper torsions (kcal/mol/rad^2)	Secondary structure (kcal/mol/rad^2)	Dummy Delta (Å)	Time (ns)
1	1.0	0.2	1.0	1.0	1.0	500.0	2	2
2	2.0	0.5	2.0	2.0	2.0	500.0	2	2
3	5.0	1.0	5.0	5.0	5.0	500.0	2	2
4	10.0	2.0	10.0	10.0	10.0	500.0	2	2
5	10.0	5.0	10.0	10.0	10.0	500.0	2	2
6	10.0	10.0	10.0	10.0	10.0	500.0	2	2
7	10.0	20.0	10.0	10.0	10.0	500.0	2	2
8	10.0	50.0	10.0	10.0	10.0	400.0	2	2
9	10.0	50.0	10.0	10.0	10.0	300.0	2	2
10	10.0	50.0	10.0	10.0	10.0	200.0	2	2
11	10.0	50.0	10.0	10.0	10.0	100.0	1	80
